# Invasive Candidiasis in Hospitalized Patients with Major Burns

**DOI:** 10.3390/jpm12010047

**Published:** 2022-01-04

**Authors:** Yi-Ling Lin, I-Chen Chen, Jung-Hsing Yen, Chih-Sheng Lai, Yueh-Chi Tsai, Chun-Te Lu, Cheng-Yeu Wu, Wei-Szu Lin, Ching-Heng Lin, Yung-Chieh Huang

**Affiliations:** 1 Division of Plastic and Reconstructive Surgery, Department of Surgery, Taichung Veterans General Hospital, Taichung 40705, Taiwan; kitty19850209@gmail.com (Y.-L.L.); stingray1191@yahoo.com.tw (I.-C.C.); rsyen@vghtc.gov.tw (J.-H.Y.); laics@vghtc.gov.tw (C.-S.L.); vv7315@gmail.com (Y.-C.T.); ctlu119@vghtc.gov.tw (C.-T.L.); jyolz.wu@gmail.com (C.-Y.W.); 2 Department of Critical Care Medicine, Taichung Veterans General Hospital, Taichung 40705, Taiwan; 3 Institute of Medicine, School of Medicine, Chung Shan Medical University, Taichung 40201, Taiwan; 4 Department of Nursing, College of Nursing, Hung Kuang University, Taichung 433304, Taiwan; 5 Department of Medical Research, Taichung Veterans General Hospital, Taichung 40705, Taiwan; weiszu@vghtc.gov.tw; 6 Department of Public Health, College of Medicine, Fu Jen Catholic University, New Taipei City 242062, Taiwan; 7 Institute of Public Health and Community Medicine Research Center, National Yang Ming Chiao Tung University, Taipei 11221, Taiwan; 8 Department of Industrial Engineering and Enterprise Information, Tunghai University, Taichung 40705, Taiwan; 9 Department of Health Care Management, National Taipei University of Nursing and Health Sciences, Taipei 112303, Taiwan; 10 Department of Pediatrics, Taichung Veterans General Hospital, Taichung 40705, Taiwan

**Keywords:** burns, invasive candidiasis, Taiwan, NHIRD

## Abstract

Background: Invasive candidiasis (IC) is a major cause of morbidities and mortality in patients hospitalized with major burns. This study investigated the incidence of IC in this specific population and analyzed the possible risk factors. Materials and Methods: We retrospectively analyzed data from the National Health Insurance Research Database (NHIRD) of Taiwan. We identified 3582 patients hospitalized with major burns on over 20% of their total body surface area (TBSA) during 2000–2013; we further analyzed possible risk factors. Result: IC was diagnosed in 452 hospitalized patients (12.6%) with major burns. In the multivariate analysis, patients older than 50 years (adjusted odds ratio (OR) = 1.96, 95% confidence interval (CI) 1.36–2.82), those of female sex (adjusted OR = 1.33, 95% CI 1.03–1.72), those with burns on the head (adjusted OR = 1.33, 95% CI 1.02–1.73), and those with burns over a greater TBSA had higher risks of IC. Conclusion: Treating IC is crucial in healthcare for major burns. Our study suggests that several risk factors are associated with IC in patients hospitalized with major burns, providing reliable reference value for clinical decisions.

## 1. Introduction 

Burns are estimated to result in 180,000 deaths globally every year [1]. In developed countries, the survival rate of patients with major burns has improved in recent years because of advances in surgical treatment and intensive care [2,3], but patients still experience prolonged hospitalization, disfigurement, and disability. Infection is a major challenge during hospitalization in patients with major burns because the skin barrier is extensively compromised.

The incidence of systemic candidiasis among patients with burns has increased from 2.19% to 4.3% in the 1990s [4,5], to 6.3% in a 2008 report [6], and to 11% in a 2015 report [3]. Some literature has suggested that the increasing trend of opportunistic fungal infections is associated with the increasing early use of broad-spectrum antibacterial agents [3,6]. In patients with burns, a fungal infection may be associated with several factors, including age, burn size, and chronic comorbidities. *Candida* species infections have outnumbered other species of fungus in literature [7,8]. In this study, we used the Taiwan National Health Insurance Research Database (NHIRD) for a retrospective analysis, focusing on patients with severe burns who required hospitalization. This study investigated the incidence of invasive candidiasis (IC) among these patients in Taiwan and related risk factors that severe burn patients might encounter.

## 2. Materials and Methods

### 2.1. Database

Taiwan’s National Health Insurance program was established in 1995, and it currently covers 99.6% of Taiwan’s population. The NHIRD is maintained by the National Health Research Institutes of Taiwan; the NHIRD is widely used for medical research and is validated as being of high quality and as being accurate in diagnoses [9]. The identification information of every individual is encrypted before the data are publicly available for research purposes. The diagnostic codes in the NHIRD are based on the International Classification of Diseases, Ninth Revision, Clinical Modification (ICD-9-CM). Patients with major burns, defined as a burn area equal to or more than 20% of the total body surface area (TBSA) or involving the eyes or head area, are included in the list of catastrophic illnesses in the NHIRD. 

### 2.2. Study Design

**Figure 1:**
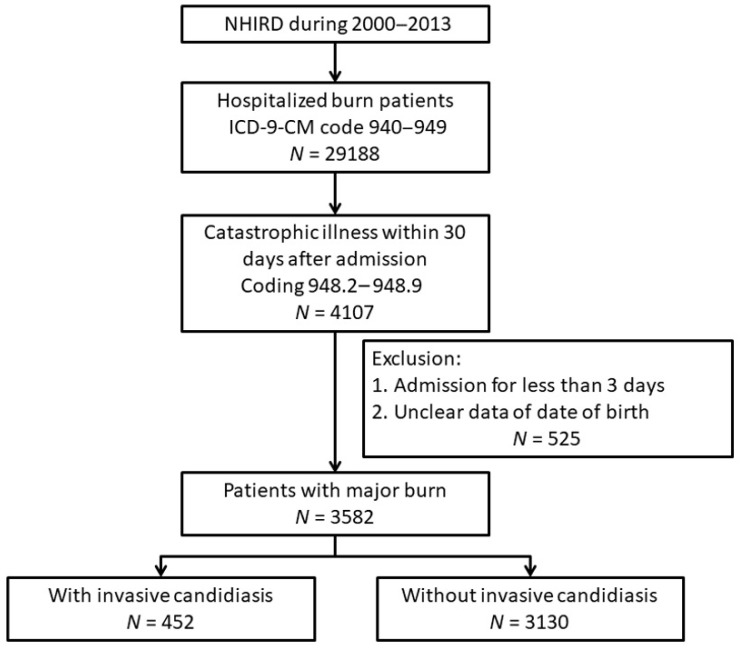
Flowchart of patient inclusion in this study. NHIRD, National Health Insurance Research Database; ICD-9-CM, International Classification of Diseases, Ninth Revision, Clinical Modification.

A flowchart of patient inclusion in this study is presented in Figure 1. In this study, we retrospectively identified all patients hospitalized with burns in the NHIRD in 2000–2013 with *ICD-9 CM* codes 940–949. To define patients with major burns, we identified patients who had been registered as having catastrophic illness within 30 days after admission, with catastrophic illness codes 948.2–948.9 (TBSA 20–29% to >90%). Patients with no registration of catastrophic illness, those with no date of birth recorded, or those who were admitted for less than 3 days were excluded from this study. The burn site was defined according to *ICD-9-CM* codes. An inhalation injury was defined as *ICD-9CM* codes 947.0, 947.1, and 947.2 [10]. The season of a patient’s hospitalization was defined as summer (May–October) or winter (November–April), with an average outdoor temperature cutoff of 24 °C.

IC was defined as a hospitalized patient with a diagnosis of ICD-9CM codes 112.5 (disseminated candidiasis), 112.81 (*Candida* endocarditis), or 112.83 (*Candida* meningitis) [11] and a prescription for systemic antifungal agents.

### 2.3. Statistical Analysis

We evaluated the patients’ baseline characteristics, including age, sex, dwelling area, family income, and chronic diseases, from the NHIRD data. We assessed the risk factors for IC among the patients with major burns by using odds ratios (ORs) and accompanying 95% confidence intervals (CIs) with a chi-squared test.

Data management and statistical analyses were performed using SAS 9.4 (SAS Institute, Cary, NC, USA). Statistical significance was indicated by a two-tailed *p*-value of <0.05.

This study was approved by the institutional review board of Taichung Veterans General Hospital (No. CE17178A-4). For privacy protection, the identities of the patients, physicians, and institutions were deidentified in accordance with the Personal Electronic Data Protection Law.

## 3. Results

From the NHIRD database for 2000–2013, we identified 3582 patients hospitalized with major burns of over 20% of TBSA, with an incidence of 0.9–1.8 in every 10,000 persons/year (Table 1). IC occurred in 452 patients, accounting for 12.6% of all patients hospitalized with major burns (annual incidence ranging from 6.58 to 17.31 per 100 patients), as presented in Table 2.

The characteristics of the patients included in our study are listed in Table 3. The distribution of age was notably different between patients with burns, and with and without IC (*p  *<  0.001); those with IC were older. Family income distributions were different between the two groups, but sex and urbanization of dwelling areas were not.

More patients with IC had inhalation injuries than did those without IC (9.1% vs. 6.5%, *p* = 0.048). The seasons of patient hospitalization were not significantly different between the two groups. The head and hands were the most common burn sites in the IC group, more common than in the non-IC group. More patients in the IC group had diabetes and hypertension than those in the non-IC group.

The 30-day mortality rates were 10.4% in the IC group and 10.2% in the non-IC group (*p* = 0.876). The 180-day mortality rates were significantly different between IC and non-IC patients (29.7% and 12.6%, *p* < 0.001).

In the multivariate analysis (Table 4), patients older than 50 years were associated with a higher risk of IC compared with those younger than 30 years (adjusted OR 1.96, 95% CI 1.36–2.82, *p* <  0.001). Male sex was associated with less IC than female sex was (adjusted OR 0.75, 95% CI 0.58–0.97, *p* = 0.029). Burns occurring on the head were also associated with IC (adjusted OR 1.33, 95% CI 1.02–1.73, *p* =  0.036). Patients with burns covering more than 30% of TBSA were also more likely to have IC compared with those with burns covering 20–29% of TBSA.

## 4. Discussion

Patients with burns are a high-risk group for invasive fungal infections [12], and fungal wound infection is independently associated with mortality in patients with burns [13]. Fungal wound infection also delays wound healing, causes prolonged hospitalization, and may promote scarring and contracture [7]. Indwelling medical devices, such as endotracheal tubes, mechanical devices, central venous catheters, urinary catheters, and possibly parenteral nutrition, are necessary for life support in patients with major burns, but they may be sources of IC. Antibiotics are usually prescribed for prolonged prophylaxis or treatment against bacteria and represent another risk factor for IC as an opportunistic infection [14].

In our study, the rate of IC among patients with major burns ranged from 6.58 to 17.31 per 100 hospitalized patients. According to recent reports, the incidence of IC has been increasing [15], but the rate has varied between different studies. In the study by Escrig et al. [14], the incidence of IC was between 0 and 46.3 per 1000 admissions. Zhou et al. reported an IC rate ranging from 6.06% to 17.54% in a single intensive care unit for burns [16]. Devrim et al. conducted a study of pediatric patients with burns and found an incidence of 11.5% for IC [17]. Additionally, exposure to spores at the burn scene when the patients tried to extinguish the fire increases the possibility of IC [18]. The improvement of laboratory techniques in the identification of *Candida* species is also related to this increasing trend [2]. The awareness and vigilance of clinicians to fungal infections could also explain a higher diagnostic rate, though this is rather difficult to measure.

The diagnosis and treatment of IC are challenging. Currently, culturing with subsequent susceptibility testing is the only diagnostic approach, but the sensitivity of this method ranges from only 21% to 71% [8,19]. Fungal infections may manifest only non-specific symptoms, and fungal cultures are time-consuming; these are some of the reasons why documenting fungal infections with cultures is difficult even when suspected [6]. Delayed antifungal administration can lead to higher mortality in hospitalization. However, prophylactic antifungal agents may lead to a higher proportion of species other than *Candida albicans* being present and a higher rate of reduced susceptibility [15]. The distributions of *Candida* species can vary with the duration of prophylaxis and the antifungal agent used for prophylaxis [19]. Other treatments including aggressive surgical debridement and catheter changes are also imperative to controlling the infection [18,20].

The risk factors vary for IC in patients with burns among different studies. Fochtmann et al. reported that female sex, gastrointestinal complications requiring surgery, non-gastrointestinal thromboembolic complications, and inhalation trauma were factors associated with proven IC [3]. According to the study by Sharma et al. [21], the incidence of fungal infection increased with age and larger TBSA involvement. In the study by Zhou et al. [16], risk factors included inhalation injury, renal dysfunction with replacement therapy, severe gastrointestinal complications, T-cell lymphopenia, and prior *Candida* colonization but not the percentage of TBSA. In our study, some risk factors were consistent with those of previous studies, such as female sex and larger TBSA involvement. Further studies with more patients may be necessary for more conclusive answers.

Female sex was found to be one of the risk factors for developing IC among patients with major burns in this study. Fochtmann and colleagues reported being female as one of the factors associated with candidemia (OR 5.03, 95% CI 1.25–24.9, *p* = 0.031) [3]. However, Zhou and colleagues reported that sex was not a significant risk factor of candidemia using multivariate logistic regression [16]. In an earlier review of patients with burns by Ha and colleagues, male sex was considered a risk factor for candidiasis (but not systemic candidiasis), with an OR of 2.4 [2]. In a report of autopsied burn patients conducted by Murray and colleagues, they found that the sex distribution was not different between patients with and without fungal infection [22]. 

Approximately 10–15% of asymptomatic women are colonized with *Candida* in their vulvovaginal areas [23]. During stress, skin and mucosa damage, urinary catheter dwelling, dysbiosis due to prolonged antibiotics use, and localized candidiasis might result in invasion candidiasis. Evidence suggested that estrogen may have an important role in *Candida* invasion and proliferation. Estrogens may inhibit *Candida*-specific human peripheral blood lymphocyte responses [24]. Estrogens also have direct effects on *Candida* cells with estrogen-binding proteins [25]. We propose that these could be part of the explanation for why the female sex is a risk factor for IC among patients with major burns. Nevertheless, further investigations are necessary to distinguish the role of sex differences in IC of patients with major burns.

The site of the burn injury was rarely reported or discussed in previous literature. We propose that burns involving the head indicate a more severe injury condition, which may not be reflected in TBSA. We excluded the effect of inhalation injury in the multivariate analysis; however, intubation and mechanical ventilation may be necessary in burn patients involving the head. Preexisting *Candida* colonization of the mucosal membranes of the nose and throat could represent a latent source of infection [26].

The mortality rate within 30 days was not significantly different between patients with burns and with and without IC; however, the mortality rate was significantly different between the two groups in 180 days. As we mentioned earlier, candidiasis acts mostly as an opportunistic infection and may not occur or be life-threatening in the early days of a burn injury. However, in long-term care, candidiasis can greatly affect patient survival. The mortality rate reported in previous literatures varied from 29% to 76% [3,11,14], but the variety could be partially explained by the different study designs (such as different definitions of major burns and exclusion criteria).

This study has some limitations. This was a retrospective database analysis study, and the patients’ physical examinations, laboratory data, *Candida* species, and doses of antifungal agents were unavailable in the database. We could not distinguish candidemia-related mortality from non-candidemia-related via the retrospect database. The population in this study might not have been large enough to reach significance in some of the statistical analyses. 

## 5. Conclusions

In conclusion, our study suggests that IC in patients hospitalized with major burns is associated with risk factors including an age older than 50 years, female sex, burns on the head, and larger TBSA involvement. Identifying high-risk patients with proper antifungal prophylaxis is paramount, and our study may provide more information for clinical decision-making.

## Figures and Tables

**Table 1:** Annual incidence of patients hospitalized with major burns (over 20% of TBSA involved).

Year	Incidence			Total Population at Risk			Annual Incidence		
							per 10^5^/Year		
	Female	Male	Total	Female	Male	Total	Female	Male	Total
2000	46	150	196	14,109,224	14,085,347	28,194,571	0.33	1.06	0.70
2001	39	127	166	13,981,319	13,896,934	27,878,253	0.28	0.91	0.60
2002	64	191	255	13,823,504	13,711,521	27,535,025	0.46	1.39	0.93
2003	94	231	325	13,651,279	13,523,811	27,175,090	0.69	1.71	1.20
2004	86	236	322	13,526,372	13,378,291	26,904,663	0.64	1.76	1.20
2005	98	241	339	13,406,980	13,244,026	26,651,006	0.73	1.82	1.27
2006	72	228	300	13,276,737	13,103,229	26,379,966	0.54	1.74	1.14
2007	82	196	278	13,154,033	12,959,788	26,113,821	0.62	1.51	1.06
2008	72	225	297	13,034,087	12,820,600	25,854,687	0.55	1.75	1.15
2009	71	187	258	12,903,363	12,664,408	25,567,771	0.55	1.48	1.01
2010	64	165	229	12,769,325	12,510,816	25,280,141	0.50	1.32	0.91
2011	52	174	226	12,666,864	12,388,276	25,055,140	0.41	1.40	0.90
2012	56	176	232	12,558,021	12,258,413	24,816,434	0.45	1.44	0.93
2013	49	110	159	12,444,261	12,125,619	24,569,880	0.39	0.91	0.65

**Table 2:** Number and annual incidence of invasive candidiasis in patients.

Year	Invasive Candidiasis			Total Number of Patients			Annual Incidence		
							per 100/Year		
	Female	Male	Total	Female	Male	Total	Female	Male	Total
2000	3	10	13	46	150	196	6.52	6.67	6.63
2001	8	10	18	39	127	166	20.51	7.87	10.84
2002	9	15	24	64	191	255	14.06	7.85	9.41
2003	16	27	43	94	231	325	17.02	11.69	13.23
2004	12	26	38	86	236	322	13.95	11.02	11.80
2005	4	27	31	98	241	339	4.08	11.20	9.14
2006	11	26	37	72	228	300	15.28	11.40	12.33
2007	12	38	50	82	196	278	14.63	19.39	17.99
2008	10	20	30	72	225	297	13.89	8.89	10.10
2009	12	28	40	71	187	258	16.90	14.97	15.50
2010	8	22	30	64	165	229	12.50	13.33	13.10
2011	9	26	35	52	174	226	17.31	14.94	15.49
2012	6	33	39	56	176	232	10.71	18.75	16.81
2013	7	17	24	49	110	159	14.29	15.45	15.09

**Table 3:** Characteristics of patients with major burns and with and without invasive candidiasis.

Characteristics	Without Invasive Candidiasis (*n* = 3130)	With Invasive Candidiasis (*n* = 452)	Total (*n* = 3582)	*p-*Value
	*n* (%)	*n* (%)	*n* (%)	
Age, mean ± SD				<0.001
<30	1146 (36.6)	99 (21.9)	1245	
30–49	1202 (38.4)	195 (43.1)	1397	
≥50	782 (25.0)	158 (35)	940	
Sex				0.376
Female	818 (26.1)	127 (28.1)	945	
Male	2312 (73.9)	325 (71.9)	2637	
Urbanization				0.301
Urban	1569 (53.7)	226 (56.5)	1795	
Suburban	432 (14.8)	48 (12.0)	480	
Rural	919 (31.5)	126 (31.5)	1045	
Family income (NTD)				0.002
≤15,840	1839 (58.8)	223 (49.3)	2062	
15,841–28,800	977 (31.2)	173 (38.3)	1150	
28,801–45,800	211 (6.7)	36 (8.0)	247	
>45,800	103 (3.3)	20 (4.4)	123	
Diabetes				<0.001
No	2973 (95.0)	410 (90.7)	3383	
Yes	157 (5.0)	42 (9.3)	199	
Hypertension				0.001
No	2812 (89.8)	382 (84.5)	3194	
Yes	318 (10.2)	70 (15.5)	388	
Hyperlipidemia				0.536
No	2991 (95.6)	429 (94.9)	3420	
Yes	139 (4.4)	23 (5.1)	162	
Burn sites				
Head	1664 (53.2)	292 (64.6)	1956	<0.001
Trunk	2196 (70.2)	326 (72.1)	2522	0.393
Upper limbs	1951 (62.3)	268 (59.3)	2219	0.213
Hands	621 (19.8)	68 (15.0)	689	0.016
Lower limbs	1726 (55.1)	233 (51.5)	1959	0.151
Internal organs	269 (8.6)	49 (10.8)	318	0.117
TBSA of burn				<0.001
20–29%	1423 (45.5)	64 (14.2)	1487	
30–39%	676 (21.6)	66 (14.6)	742	
40–49%	398 (12.7)	82 (18.1)	480	
50–59%	187 (6.0)	62 (13.7)	249	
60–69%	125 (4.0)	57 (12.6)	182	
70–79%	106 (3.4)	50 (11.1)	156	
80–89%	94 (3.0)	37 (8.2)	131	
90% or more	121 (3.9)	34 (7.5)	155	
Inhalation injury				0.048
No	2925 (93.5)	411 (90.9)	3336	
Yes	205 (6.5)	41 (9.1)	246	
Season				0.675
Winter	1449 (46.3)	214 (47.3)	1663	
Summer	1681 (53.7)	238 (52.7)	1919	
Mortality				
In 30 days	318 (10.2)	47 (10.4)	365	0.876
In 180 days	404 (12.6)	134 (29.7)	538	<0.001

**Table 4:** Multivariate analysis of factors associated with invasive candidiasis.

Variables	Multivariate	*p-*Value
	Adjusted OR (95%CI)	
Age, mean ± SD		
<30	1.00 (ref)	
30–49	1.34 (0.97–1.84)	0.074
≥50	1.96 (1.36–2.82)	<0.001
Gender		
Female	1.00 (ref)	
Male	0.75 (0.58–0.97)	0.029
Urbanization		
Urban	1.00 (ref)	
Suburban	0.89 (0.63–1.27)	0.517
Rural	1.03 (0.80–1.32)	0.838
Family income (NTD)		
≤15,840 TWD	1.00 (ref)	
15,841–28,800 TWD	0.98 (0.74–1.29)	0.865
28,801–45,800 TWD	1.19 (0.76–1.85)	0.446
>45,800 TWD	1.13 (0.64–2.01)	0.674
Diabetes	1.59 (0.99–2.54)	0.053
Hypertension	1.17 (0.80–1.71)	0.421
Hyperlipidemia	0.81 (0.47–1.41)	0.456
Burn sites		
Head	1.33 (1.02–1.73)	0.036
Trunk	0.79 (0.60–1.05)	0.103
Upper limbs	0.83 (0.64–1.07)	0.149
Hands	0.80 (0.59–1.09)	0.154
Lower limbs	0.88 (0.69–1.12)	0.301
Internal organs	0.89 (0.40–1.98)	0.776
TBSA of burn		
TBSA 20–29%	1.00 (ref)	
TBSA 30–39%	2.41 (1.63–3.57)	<0.001
TBSA 40–49%	4.81 (3.28–7.05)	<0.001
TBSA 50–59%	8.51 (5.63–12.84)	<0.001
TBSA 60–69%	11.59 (7.45–18.05)	<0.001
TBSA 70–79%	14.02 (8.83–22.25)	<0.001
TBSA 80–89%	12.06 (7.28–19.97)	<0.001
TBSA 90% or more	6.91 (4.04–11.82)	<0.001
Inhalation injury		
No	1.00 (ref)	
Yes	1.16 (0.48–2.76)	0.744
Season		
Winter	1.00 (ref)	
Summer	0.83 (0.66–1.04)	0.111

## Data Availability

Raw data for this work were obtained by application from the National Health Insurance Research Database, Taiwan (http://nhird.nhri.org.tw/en/index.html, accessed on 19 July 2020), and may not be shared according to the database’s rules governing use. Access to the data used in this study may be obtained by citizens of Taiwan who fulfill the requirements of conducting research projects.
